# Dieback and dredge soils of *Phragmites australis* in the Mississippi River Delta negatively impact plant biomass

**DOI:** 10.1038/s41598-024-52488-4

**Published:** 2024-01-22

**Authors:** Herie Lee, Rodrigo Diaz, James T. Cronin

**Affiliations:** 1 Department of Biological Sciences, Louisiana State University, 202 Life Sciences Building, Baton Rouge, LA 70803 USA; 2 Department of Entomology, Louisiana State University, Baton Rouge, LA 70803 USA

**Keywords:** Restoration ecology, Wetlands ecology

## Abstract

*Phragmites australis* is exhibiting extensive dieback in the Lower Mississippi River Delta (MRD). We explored the potential for restoration of these marshes by (1) characterizing the chemical profiles of soils collected from healthy and dieback stands of *P. australis* and from sites recently created from dredge-disposal soils that were expected to be colonized by *P. australis* and (2) experimentally testing the effects of these soil types on the growth of three common *P. australis* lineages, Delta, Gulf and European. Soil chemical properties included Al, Ca, Cu, Fe, K, Mg, Mn, Na, P, S, Zn, % organic matter, % carbon, % nitrogen, and pH. Dieback soils were characterized by higher % organic matter, % carbon, % nitrogen, and higher S and Fe concentrations, whereas healthy soils had higher Cu, Al, P and Zn. In comparison, dredge sites were low in nutrients and organic matter compared to healthy soils. Rhizomes of each *P. australis* lineage were planted in each soil type in a common garden and greenhouse and allowed to grow for five months. Aboveground biomass was 16% lower in dieback and 44% lower in dredge soils than in healthy soils. However, we could detect no significant differences in response to soil types among lineages. Although dredge and dieback sites are not optimal for *P. australis* growth, plants can thrive on these soils, and we recommend restorative measures be initiated as soon as possible to minimize soil erosion.

## Introduction

Coastal wetlands are among the most productive and valuable ecosystems on the planet^1,2^. These essential ecosystems provide important services such as sediment stabilization and storm surge protection by tall-statured grasses and mangroves^3^, removal of excess nutrients from the water column^4^, pollutant trapping^5^, CO_2_ and CH_4_ sequestration^6^, and support of nursery habitats and feeding grounds for wildlife^7^. However, coastal wetlands are severely threatened by changing climatic, oceanographic, and ecological conditions, as well as anthropogenic activities^8^.

The perennial marsh grass, *Phragmites australis* (Cav) Tren. ex Steud, which frequently dominates freshwater and brackish marshes worldwide, has exhibited extensive dieback in the Lower Mississippi River Delta (MRD) dating back to about 2015^9–11^. Dieback syndrome of *P. australis* has been characterized as the retreat of plants from deep water, increased stem clumping within a stand, stunted stem growth, premature senescence of leaf tissue, and high occurrences of dead or decaying rhizomes^12–15^. The ongoing dieback of *P. australis* is of serious ecological and economic concern in the rapidly deteriorating coastal wetlands because it may expedite land conversion by reducing sediment trapping and consequentially allowing the infilling of essential navigation channels^10,16^. Moreover, vegetation dieback events are often precursors to land loss because they result in the conversion of marshes into bare mudflats or open water^1^. Land loss has been a well-established problem in coastal Louisiana and considerable effort and expense have been invested in creating new wetlands from dredge materials^17^.

Multiple abiotic and biotic stressors have been implicated in the dieback of *P. australis* in Europe^13,18^, China^19^ and the USA^10,20–22^. These factors include eutrophication coupled with prolonged waterlogging, elevated sulfide concentrations and organic matter accumulation, mechanical damage by waves or herbivores and fungal pathogens^13,14,18,23–25^. In flooded and waterlogged marsh soils, sulfur reduction leads to the production and accumulation of hydrogen sulfide, which can cause lignification and blockages in the plant’s vascular system, weakened stems, and dieback symptoms of *P. australis*^12^. Accumulation of phytotoxins such as hydrogen sulfide can negatively impact the reed’s ability to efficiently uptake and assimilate nutrients for energy and growth^12^. Additionally, Furtig et al.^26^ concluded that accumulation of heavy metals (e.g., copper, iron) in belowground organs might have induced reed die-back in European lakes. In Italy, Gigante et al.^27^ observed reduced growth of *P. australis* at elevated concentrations of copper, zinc, nickel, total chromium, cobalt and iron. As a result of dieback, decay and decomposition of large quantities of damaged *P. australis* and organic matter may produce and release organic acids, sulfides and heavy metals^18^. In many cases, it is unclear whether soil conditions reported following a dieback event are a direct cause or consequence of dieback.

A complicating factor in the dieback of *P. australis* in the MRD is that this region is composed of several phenotypically and phylogenetically distinct lineages of *P. australis,* including those of native and nonnative origin. In the MRD, the dominant lineage is Delta (haplotype M1) and is of North African and Mediterranean origin^10,29,30^. A second common lineage is of Eurasian origin (haplotype *M*; hereafter, the EU lineage) and is regarded as invasive across much of North America^31^. In the MRD, EU occurs in scattered monodominant stands that border larger Delta stands^10,29^. Lastly, the Gulf Coast lineage (haplotype *I* or subspecies *P. australis berlandieri*), also known as Land-type, is widespread throughout Central and South America^29,31^. In North America, it is found throughout the Gulf Coast to the West Coast and inhabits more elevated areas in the MRD^29,31,32^. At present, it is unknown whether Gulf was introduced to the United States or expanded naturally from Central America. The Delta lineage, which accounts for > 95% of the *P. australis* in the MRD, appears to be the most susceptible to dieback^10,21,22^. Although we have observed a couple of instances where Delta recolonized dieback sites, most areas have converted to bare mudflats, open water or have been replaced by opportunistic plants such as *Colocasia esculenta* L. (elephant ear), *Alternanthera philoxeroides* (Mart.) (alligatorweed), or *Pontederia crassipes* (Mart.) (water hyacinth)^22,33^.

The purpose of our study was (1) to investigate the differences in soil chemistry between stands of healthy *P. australis*, stands where *P. australis* is currently experiencing dieback and sites newly constructed from dredge soils; and (2) assess whether these different soil types affect the growth of different *P. australis* lineages. Our intent was not to disentangle the chemical changes leading to dieback from those resulting from dieback. Instead, we focused on whether those dieback soils would limit *P. australis* growth and recovery following dieback. In the Winter and Spring of 2019, soils were collected from replicate healthy, dieback and dredge sites in the MRD and analyzed for their chemical composition. Subsequently, we conducted greenhouse and common garden experiments to assess whether soil type, *P. australis* lineage (Delta, Gulf, EU) and their interaction influenced *P. australis* aboveground growth (biomass, stem height and stem density). We tested the following predictions: (1) dieback soils have the highest concentrations of organic matter, sulfur, and heavy metals, whereas dredge soils have the lowest nutrient and organic content levels; (2) plants potted in dieback soils produce less biomass than plants potted in healthy soils; (3) nutrient-poor dredge soils yield plants with the lowest biomass; (4) the Delta lineage, which appears more at risk to dieback^21,22^, will be most negatively affected by dieback soils; and (5) as a successful invader of a wide array of environmental conditions, the EU lineage of *P. australis* outperforms the other lineages in dieback and dredge soils.

## Materials and methods

### Study system

The Mississippi River is the primary source of freshwater, nutrients, and sediments to the Gulf of Mexico, as well as the world’s 7th largest river in discharge^34,35^. Since the twentieth century, the lower MRD has been subjected to increased inputs of nutrients, contaminants and metals (e.g., Fe, Mn, Cu) from runoff containing agricultural pesticides, fertilizers and industrial byproducts (e.g., PCBs, dioxins/furans, petroleum)^36^ leading to eutrophic conditions in some cases^37^. Additionally, saltwater intrusion and acidification, driven by natural (e.g., eustatic sea-level rise, storm surges, hurricanes) and anthropogenic (e.g., land drainage, man-made water diversions) factors, and a warming climate, have also contributed to changes in this wetland landscape^8^.

Since 1976, the United States Army Corps of Engineers, New Orleans District, has created approximately 63 km^2^ of coastal land through dredge sediment diversions in the lower MRD^17^. There are plans for an additional 79 restoration projects that include wetland creation, sediment diversions, and barrier island restoration in the Lower MRD^38^. One objective with river sediment diversions is to increase wetland area by creating new and sustainable wetlands^37^. Although we have observed natural colonization of *P. australis* in newly constructed wetlands along the lower MRD, there are no known reports of the extent of this colonization.

*Phragmites australis* is a cosmopolitan perennial grass, common in a wide range of wetland ecosystems, including fresh and brackish wetlands, lake and pond margins, disturbed wetlands, wet meadows and ditches^39^. *Phragmites australis* forms dense monospecific stands growing up to 3–5 m tall^40^. The tallest among the lineages is Delta with stems reaching 4–5 m in height^10^. The introduced EU lineage is less abundant than the Delta lineage in the Lower MRD, but has high biomass productivity, prolific seed production and increased rhizome production which enables it to outcompete other plant species, including other *P. australis* lineages^41^. Salinity-induced stress may contribute to the dispersion of *P. australis* lineages with the Gulf lineage being the least salinity tolerant^42^ which may explain why it is limited to levees and high-elevation embankments within the MRD^22^.

### Healthy, dieback, and dredge soil chemistry

To determine whether soils from dieback, healthy and dredge sites have different chemical profiles, soils were collected in the Winter and Spring of 2019 from within the Pass-a-Loutre Wildlife Management Area in the Lower MRD. The first collection of soils was made on January 9, 2019 from three separate healthy and dieback sites (“Winter Survey; See Supplementary Material 1, Table S1a). The second collection was made on May 29, 2019 from two healthy, two dieback and two dredge-disposal sites (“Spring Survey”; Table S1b). All of these sites were either in standing fresh water or bordering fresh-water channels (< 1 ppt NaCl). Candidate sites were preselected from maps of the reported change in NDVI between 2008 and 2017^11^ and stand status and health was confirmed by expert knowledge of Louisiana Department of Wildlife and Fisheries personnel. Sites categorized as healthy had minimal NDVI change in the previous three years and at the time of survey had dense stands of live *P. australis* stems often spanning > 1 ha in size. Sites categorized as dieback were from the highest category of dieback NDVI change (i.e., greatest decline in NDVI between 2016 and 2019). On-site inspection of those stands clearly revealed the telltale symptoms of dieback^13,14^: stunted growth of stems, premature senescence of leaf tissue, the presence of dead meristematic tissue, high patchiness in the distribution of plants, few new live stems and extensive open spaces. All non-dredge sites were located along navigable channels and had water depths ranging from 20 to 50 cm at the time of the survey.

The two dredged sediment locations were selected within the Pass-a-Loutre WMA of the Head of Passes Hopper Dredge Disposal Area (HDDA) created by the U.S. Army Corps of Engineers^17^ (Table S1b). The first site was from South Sawdust Bend that had 46.5 ha of dredge sediment discharged approximately 6 months prior to collection^17^. The second site was from North Sawdust Bend that had 29 ha discharged approximately 1 year prior to collection^17^. Sediment material was dredged upriver and hydraulically pumped out at both locations. We note that at both sites, *P. australis* already had begun to establish during the collection trip on May 29, 2019.

At each of three locations per site (separated by at least 5 m), we excavated 19 L of soil. We dug to depth of 50 cm below the soil surface and discarded the top 10 cm (predominantly comprising coarse organic material). For the dredge sites, soils were collected approximately 50 m from the water’s edge in open areas free of vegetation. The three soil samples from each site were combined and separate soil collections were made for the Winter-Spring and Spring–Summer plant-biomass experiments. At the same time, we also collected an additional ~ 250 g of soil from each of the three locations per site, placed each sample in a separate plastic bag and transported the bags on ice to the laboratory. Samples were stored at 4 °C until soil chemistry tests were performed.

The characterization of the soil chemical properties was performed by the LSU AgCenter Soil Testing and Plant Analysis Lab. For each soil sample, concentrations of Ca, Cu, K, Mg, Na, P, S, and Zn (ppm) were determined using Mehlich 3 as the extractant in 2 g soil with 20 mL solution of 3.75 M NH_4_F–0.25 M EDTA NH_4_NO_3_, CH_3_COOH, and HNO_3._ Concentrations of Fe and Mn (ppm) were determined using 10 g of soil with 20 mL of pH 7.3 and 0.005 M diethylenetriaminepentaacetic acid solution (DTPA). Al (ppm) was estimated from 2 g of soil with 20 mL solution of 0.1 M of BaCl_2_/NH_4_Cl. Analytes in all extracts were determined Inductively Coupled Plasma Optical Emission Spectroscopy (ICP—OES). A pH meter and electrode were used to measure the pH of the supernatant obtained from the mixture of 10 g of soil with 10 mL of deionized H_2_O. Soil organic matter (OM), as a percentage, was determined from 1 g of soil using an acid–dichromate oxidation solution of 10 mL of 0.1 N potassium dichromate (K_2_Cr_2_O_7_), 20 mL of concentrated sulfuric acid (H_2_SO_4_), and 90 mL of H_2_O and analyzed using a Dip-Probe colorimeter. Lastly, % C and % N was measured by dry combustion using a LECO Carbon/Nitrogen Dumas Analyzer. Methods are summarized in Table S2.

### Soil type and lineage effects on *P. australis* growth

To test whether soil type influenced *P. australis* growth parameters, we conducted two separate experiments in Baton Rouge, Louisiana, USA using the soils from the Winter and Spring soil surveys.

#### Winter–Spring experiment

On January 10, 2019, a greenhouse experiment was initiated with soils collected the previous day from Pass-a-Loutre WMA (Winter soil survey). The Winter-Spring Experiment was conducted in the greenhouse because the greenhouse had moderate temperatures at the time of planting (20–33 C). Rhizome cuttings from each of six source populations of *P. australis* (3 Delta, 1 EU, and 2 Gulf; Table S3a) were planted in each of two soil types (Table S1a, dieback and healthy) in 2.6 L pots. We originally intended to have two representative populations for each lineage; however, a Delta population (PLM) was initially misclassified as EU. For every *P. australis* source population × soil type combination, we had 7–8 replicates for a total of 265 pots. Rhizome cuttings from each source population were obtained from a common garden that has been maintained at Louisiana State University since 2010^28^. The populations used for this study (Table S3a) had been growing under identical conditions in the garden for at least two years; thus, maternal environmental effects on plant growth metrics were likely to be minimal. Gulf populations originated from High Island, TX (HI) and Bayou Sauvage National Wildlife Refuge (SAU) in St. Tammany Parish, Louisiana.

Harvested rhizome material was rinsed to remove all sediment and cut into single fragments (15–20 g wet weight with at least one node). A single rhizome cutting was inserted upright into a 10.2 × 10.2 cm square plastic nursery pot (14 cm tall) and filled with either dieback or healthy soil. Each pot was placed into a separate 23 × 23 × 8 cm clear plastic tray filled with water. Separate watering trays were used to avoid movement of soil nutrients/contaminants between pots.

Pots were watered with tap water as often as needed by wetting the soil surface and filling the trays. On 4 March, we added 5 ml fertilizer solution to each pot. The fertilizer comprised a mixture of 45 g of Miracle Gro (24-8-16 NPK, The Scotts Miracle-Gro Company®, Marysville, Ohio) and 132 ml of Liquinox® (iron and zinc supplement; Liquinox Co., Orange, California) and diluted in 11.3 L of water. Because the purpose of this study was to evaluate plant growth in these different soils, we only provisioned the plants with a small inoculum of nutrients at the start of the experiment to ensure that plants had at least a short-term source of resources to initiate growth^22^. Approximately five weeks later, pots that had no growth were repotted with fresh rhizome material from the same plant source population.

Five months post-planting, on June 11, 2019, the experiment was terminated, and plants were harvested before the plants became pot bound. We measured the height of the tallest stem per pot (stem base to the tip of the uppermost green leaf, in cm) and number of stems per pot. Afterward, all stems per pot were cut at the base and transferred to a paper bag to dry in the greenhouse. Starting at 30 d post-harvesting, we measured the mass of a subset of 20 bags of plant material every 3–5 d. When plant mass no longer changed, we recorded the final dry mass. Because aboveground biomass is most closely linked to NDVI^11^, we focused primarily on this growth metric; although, we report how changes in stem density and stem height contributed to the change in biomass in the Supplementary Materials (Table S7, Fig. S4).

#### Spring–Summer experiment

On May 30, 2019, we repeated the above experiment using the dieback, healthy, and dredge soils from the Spring soil survey (Table S1b). The Spring–Summer experiment was conducted in an outdoor common garden at Louisiana State University where temperatures ranged from night-time lows of 21 C to day-time highs of 36 C. Sources of *P. australis* were limited to two Delta and two EU populations (Table S3b). For Delta, we used two of the three population sources used in Winter-Spring experiment (Earl3, Earl4; Table S3b). For EU populations, we used TELM, originally sourced from Missouri and Earl2 from the Lower MRD (Table S3). The Gulf lineage was excluded from this experiment because there was insufficient material available from the common garden. There were four replicates of each plant population × lineage × soil type for a total of 288 potted plants. In this experiment, plants potted with the same soil source were placed in 1.2 m diameter plastic pools filled with tap water to a depth of ≈ 15 cm. Twice weekly, water was added to the pools to maintain a depth of ≈ 15 cm. Six months later (December 5, 2019), the experiment was terminated, and we repeated the harvesting procedure outlined above.

Pools were watered twice a week, or as needed. After one month, we added 236 mL of the Miracle Gro—Liquinox solution to each pool (an equivalent amount to what was added to each individual pot in the previous experiment).

### Data analysis

#### Soil chemistry analysis

Our first objective was to assess whether soil types differed in their chemical profiles. To accomplish this, we first reduced the dimensionality in the data with principal component analysis (PCA). Separate PCAs were conducted for each survey period using R (R Team, 2021) and function *prcomp*. PCA was used to reduce the 15 chemical elements to a new set of independent (canonical) eigenvectors or principal components (PC). Separate PCAs were performed for the Winter and Spring survey datasets because dredge soils were only collected in the latter time period. Soil chemical PCs were retained for analysis if their eigenvalues were > 1^44^. To determine if the different soil types can be differentiated based on their soil chemical profiles, we next performed linear discriminant analysis (LDA) using the retained PCs as independent variables. LDA computes directions, called linear discriminants (LDs), that represent the axes that maximize the separation between multiple classes (e.g., soil types). To evaluate the prediction performance of the model, datasets were split into a training set (75%) and a test set (25%). A posteriori analysis of correct classification was done using the *predict* function available in the *MASS* package. Lastly, after documenting that chemical profiles differed among soil types, we conducted separate *t*-tests for the Winter dataset (healthy vs. dieback) and one-way ANOVA for the Spring dataset (healthy, dieback, dredge) to determine whether specific chemical elements or compounds differed significantly among soil types. To minimize the risk of type I errors associated with multiple non-independent tests, we used Bonferroni-corrected levels of α for determining statistical significance. Pairwise comparisons tests for the Spring dataset were determined using Tukey’s HSD test. All statistical analyses were performed in RStudio using the packages *lda* and *MASS*.

#### *Analysis of soil type and lineage effects on *P. australis* growth*

Due to differences in soil types and *P. australis* lineages used and rearing methods (plants grown in individual trays in the Winter-Spring experiment versus grouped by soil type in pools in the Spring–Summer experiment), separate statistical analyses were performed for each time period. We conducted generalized linear mixed model (GLMM) analyses to test whether individual growth metrics were influenced by soil type, *P. australis* lineage and their interaction. Soil collection site, *P. australis* population and pool number (Spring–Summer experiment only) were treated as random effects to account for location effects within the MRD, within-lineage variation, and position effects within the garden plot, respectively. Model assumptions were assessed by visually inspecting residual plots and quantile–quantile plots. Goodness-of-fit was calculated using package *MuMIn*^45^. This package reports the marginal *R*^2^, which provides the variance explained by all fixed effects, and the conditional *R*^2^ which provides the variance explained by the entire model with fixed and random effects combined. Marginal means ± SE for each treatment or treatment combination were computed using *ggemmeans*. Contrasts between pairs of means were assessed using package *eemeans*, where *P* values were adjusted using Tukey’s method. We performed all statistical analyses in R^43^ with packages *lme4*, *MuMIn* and *ggemmeans.*

## Results

### Soil chemistry analysis

#### Winter survey

Based on our multivariate analysis of the chemical profiles of healthy and dieback soils, the first principal component accounted for 61.4% of the total variation and had negative loadings for most of the chemical variables except for Al, Cu, P, and pH, which had positive loadings (i.e., the weights for each variable) (Fig. S1a). The second PC accounted for 15.9% of the total variation and had high negative loadings for Cu (*r* = − 0.58; contribution = 33.3%) and Al (*r* = 0.54; 29.2%) and thus appeared to be a factor for metals (Fig. S1b). The third PC accounted for 8.7% of the total variation and was moderately correlated with Ca (*r* = 0.45; 20%), K (*r* = 0.45; 20%) and P (*r* = 0.36; 16%) and negatively correlated with Al (*r* = − 0.40; contribution = 16%) (Fig. S1c). We interpret PC3 as a factor associated primarily with soil mineral/nutrient content.

**Figure 1:**
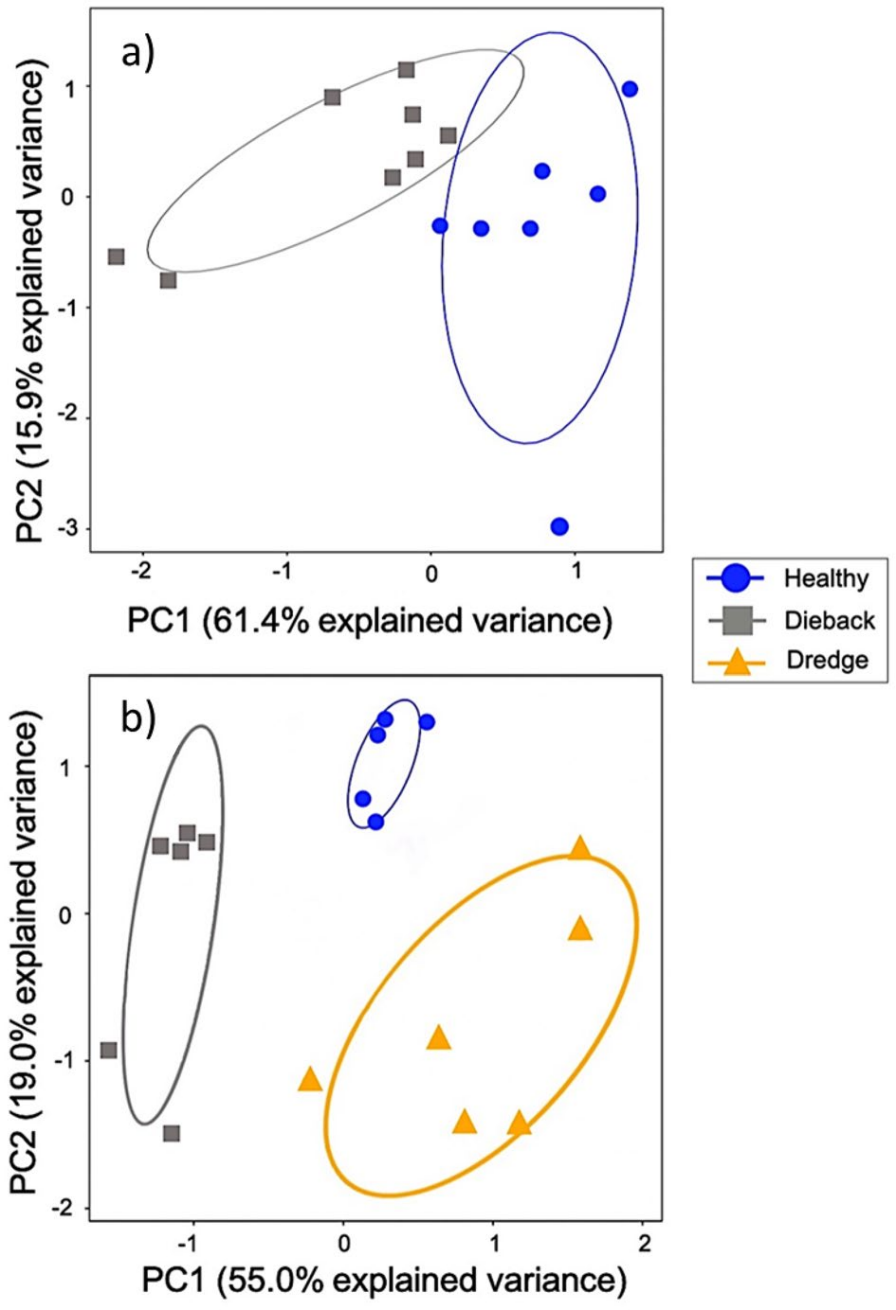
Variability explained by the first two axes (correlation biplot) of the distribution of 15 chemical variables measured in (**a**) the Winter Soil Survey and (**b**) the Spring Soil Survey. The proportion of variability explained by each PC is identified in the axes labels.

There was a strong separation of healthy and dieback soils as demonstrated in the biplot of PC1 and PC2 (Fig. 1a) and this was confirmed by the LDA. The LDA model was 100% correct in assigning soil samples to healthy or dieback sites (Table 1a). In fact, linear discriminant 1 (LD1) alone significantly differentiated between dieback and healthy soils (*t* = 3.9 *P* < 0.05).

**Table 1:** Prediction accuracy results for classifying soil types using Fisher’s Linear Discriminant Analysis (LDA) for (a) Winter (dieback, healthy) and (b) Spring (dieback, dredge, healthy) soil surveys. The first three principal components (PC) scores derived from the Principal Component Analysis (PCA) were used as the explanatory variables.

(a) LDA-classified Winter survey				(b) LDA-classified Spring survey				
Predicted	Dieback	Healthy	Sum	Predicted	Die-back	Dredge	Healthy	Sum
Dieback	8	0	8	Die-back	6	0	0	6
Healthy	0	7	7	Dredge	0	6	0	6
				Healthy	0	0	5	5
Overall accuracy			100%					100%

Many of the chemical characters considered in our study were strongly correlated with one another (Table S4a). Soil mineral nutrient content (%C, %N) was strongly positively correlated with organic matter content. Organic Matter content, % C, and % N were all strongly positively correlated with each other (*R* ≥ 0.97, *P* ≤ 0.001; Table S4a). Additionally, % OM, % C, and % N were positively correlated with Ca, Fe, K, Mg, Mn, Na, S and Zn, whereas pH and P content were strongly negatively correlated with the same properties (Table S4a). Because of these correlations, it was not surprising that suites of chemical characters exhibited similar relationships with soil type. In relation to healthy soils, dieback soils had significantly higher OM (47%), C (60%), and N (62%), Fe (56%), Mg (47%) and Na (46%) (Table S5). Dieback soils also had higher concentrations of K (11%), Mn (76%), Zn (20%) and S (75%) than healthy soils, but the differences were not statistically significant (Table S5). Dieback soil pH ranged from 4.27 to 6.93 and averaged 5.8 ± 0.27 (*n* = 8) and healthy soil pH ranged from 7.18 to 7.67 and averaged 7.40 ± 0.06 (*n* = 7) (*t* = -6.55, *P* < 0.05). In contrast, healthy soils had higher concentrations of Cu (39%), Al (78%), Ca (63%) and P (19%) but the differences were only significant for Cu and Al (Table S5).

#### Spring survey

The first three principal components had eigenvalues > 1 and combined to explain 82.7% of the total variance in the soil chemical properties tested (Fig. 1b). PC1 accounted for 56.6% of the total variance and was weakly negatively loaded with pH and positively loaded with all other variables (Fig. S2a). PC2 explained 20.3% of the total variation and had moderately negative loadings by Fe (*r* = − 0.44; contribution = 19.7%) and Al (*r* = − 0.39; 15.5%) and moderately positive loadings P (*r* = 0.42; 18.0%) and Ca (*r* = 0.38; 14.7%) (Fig. S2b). Finally, PC3 explained 8.7% of the total variation and was moderately and negatively loaded with Mn (*r* = 0.57; 32.0%) and positively loaded with S (*r* = 0.58; 34.1%) (Fig. S2c). The LDA model using these PCs successfully discriminated 100% of all soil samples to their respective soil type (Table 1b). The first linear discriminant (LD1) explained 82% of the variance, and the second linear discriminant (LD2) explained 18% of the remaining variance (Table 1b).

Correlations among soil chemical characteristics were comparable to those findings for the winter survey (Table S4b). Similar to our findings with the Winter soils, dieback soils had higher %OM (45%), %C (70%), and %N (83%) than healthy soils (Table S6), supporting our prediction 1. Dieback soils also had higher concentrations of Al (31%), Fe (44%), Mg (25%), Mn (11%), Na (25%), S (14%); however, only for S was the difference between soil types significant (Table S6). In contrast, healthy soils had higher concentrations of Ca (32%), Cu (31%), K (5%), P (26%), and Zn (3%) than dieback soils (only the former two were statistically significant). Finally, the pH of dieback soils was more acidic and averaged 6.41 ± 0.09 pH compared to healthy soils with an average of 7.43 ± 0.06 (Table S6).

Among the three soil types, dredge soils exhibited comparably lower concentrations of Ca, Cu, Fe, K, Mg, Mn, Na, %OM, S, Zn, and % C (Table S6). Notably, dredge soils had 7.2 and 4.0 times lower % OM than dieback and healthy soils, respectively. Finally, pH was 7.71 in dredge soils, 20.2% and 3.9% higher than in dieback or healthy soils, respectively (Table S6).

### Soil type and lineage effects on *P. australis* growth

#### Winter–Spring experiment

**Figure 2:**
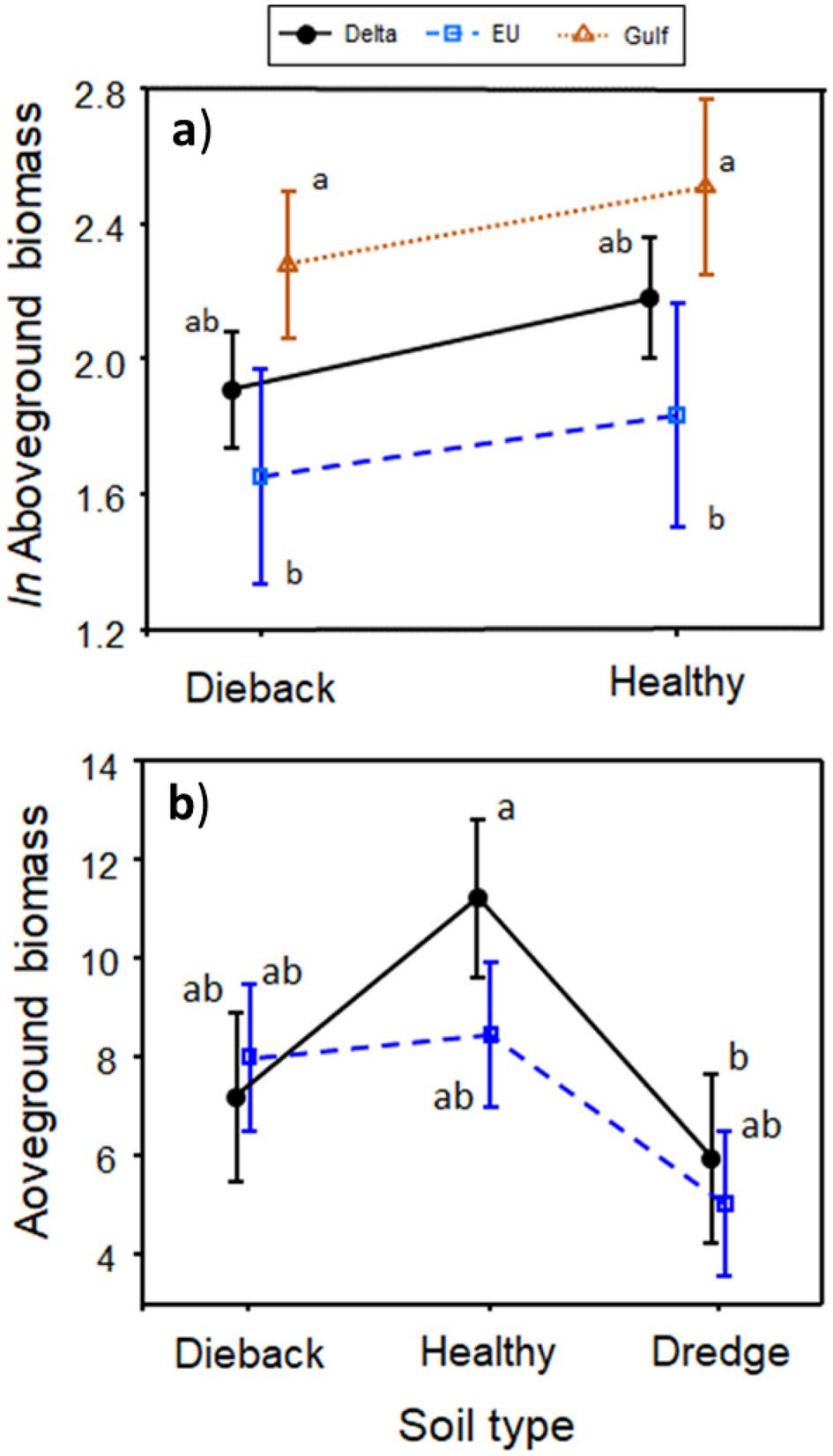
The effects of soil type (dieback, healthy, and dredge) and *P. australis* lineage on aboveground biomass for (**a**) the Winter–Spring experiment and (**b**) the Spring–Summer experiment. Reported values are marginal means ± SE. Different letters between groups indicate significant differences at *P* ≤ 0.05.

We predicted that biomass for all three *P. australis* lineages would be lower when grown in dieback as compared to healthy soils but that the Delta lineage would be most negatively affected by dieback soils than the EU lineage (prediction 4). Plants grown in dieback soils, irrespective of their lineage, had an average of 16.3% lower biomass (based on back-transformed estimates of the marginal means of *ln* biomass) than those grown in healthy soils (*t* = − 3.20, *P* = 0.02, Table 2a, Fig. 2a). The loss of biomass in dieback soils appears to be related to changes in stem production and not stem height (Table S7a). We found a 20% reduction in stem counts when plants were grown in dieback relative to healthy soils (healthy: 8.45 ± 1.31, dieback: 7.07 ± 1.10; *t* = − 2.531, *P* = 0.032; Fig S3a) but no significant change in maximum stem heights between the two soil types (*t* = − 0.521, *P* = 0.6197; Table S7b and Fig. S3b).

**Table 2:** Separate generalized linear mixed model results for the effects of soil type (dieback, dredge, healthy), *P. australis* lineage (Delta, EU, Gulf), and all possible interactions on (a) *ln* biomass for the Winter–Spring experiment and (b) biomass for the Spring–Summer experiment. Sources of variation with an * indicate significance (*P* ≤ 0.05).

	Sums of squares	*df* (num)	*df* (denom)	*F*	*P*-value
(a) Winter–Spring experiment					
Lineage	0.43	2	2.99	1.52	0.351
Soil type	1.56	1	228.2	10.95	0.001*
Lineage × soil type	0.18	2	228.23	0.62	0.541
(b) Spring–Summer experiment					
Lineage	1.87	1	2.05	0.288	0.644
Soil type	71.83	2	17.46	5.54	0.014*
Lineage × soil type	46.58	2	113.71	3.59	0.031*

Contrary to our fourth prediction, we found no evidence that the Delta lineage performed differently than the other two lineages when grown in the two soil types (i.e., no soil type × lineage interaction; Fig. 2a). Moreover, prediction 5, that the invasive EU lineage would outperform the other two lineages in dieback soils, was also unsupported (Fig. 2a). Overall, the fixed effects in the GLMM explained 22.3% of the variation in the model (based on the marginal *R*^*2*^*),* whereas the combined fixed and random effects (associated with different soil sources and rhizome sources) explained 56.7% of the model variation (based on the conditional *R*^*2*^).

#### Spring–Summer experiment

For the Spring–Summer experiment, 50% of the potted rhizome cuttings failed to survive to the end of the study period (148 of 288 pots). Based on a logistic regression analysis (where the response variable was live or dead), there was a significant difference in likelihood of survival between lineages (*z ratio* = − 7.40, *P* < 0.001). Delta and EU plants had 24.3% and 72.9% survival, respectively. The cause of the high mortality in the Delta lineage in the Spring–Summer experiment remains unknown. However, there was no significant difference in survival between plants grown in healthy and dieback soils (*z ratio* = − 0.36, *P* = 0.932) nor a lineage × soil type interaction (*z ratio* ≥ − 0.34, *P* ≥ 0.32). Consequently, replacement pots were equally distributed between soil types and it is unlikely that these replacements would affect our results regarding soil type. Lastly, for the model involving biomass, the fixed effects explained 21% and the full model explained 60% of the variation in biomass.

Using only plants that were alive at the end of the study, we found similar results to the early-season experiment. Biomass was significantly influenced by soil type but also the soil type × lineage interaction (Table 2b and Fig. 2b). Consistent with predictions 2 and 3, plants grown in dieback and dredge soils had 23% and 44% lower biomass, respectively than plants grown in healthy soils (Fig. 2b); although the difference was only significant for the dredge soils. Only the Delta lineage exhibited a significant decrease (47%) in biomass between healthy and dredge soils (Fig. 2b).

In the later-season experiment, stem counts for plants grown in healthy soils were 23% and 35% higher than for plants grown in dieback and dredge soils, irrespective of *P. australis* lineage (*t* = − 3.16, *P* = 0.012; *t* = − 4.64, *P* < 0.001; Table S7c and Fig. S3c). EU plants were particularly affected by soil type (significant soil type × lineage interaction)—stem counts were reduced by 28% and 38% in dieback and dredge relative to healthy soils (*t* = − 4.16, *P* = 0.005; *t* = − 5.69; *P* < 0.001, respectively; Table S7d and Fig. S3d). For Delta, the difference in stem counts among the soil types was not statistically significant. Finally, stem heights were unaffected by lineage or soil type (Fig. S3d).

## Discussion

Our study suggests that the soil chemistry of dieback sites 1–2 years following drastic reductions in standing biomass based on normalized difference vegetation index (NDVI) measurements^11^ is markedly different from healthy sites and that these soil conditions may significantly hinder *P. australis* recovery. Between our two greenhouse/common-garden experiments, dieback soils reduced aboveground biomass of *P. australis* by 16% and 23% relative to plants grown in healthy soils, however the difference was only significant for the Winter-Spring experiment. Marsh habitats constructed from dredge material require vegetation like *P. australis* to stabilize sediments; however, they can be nutritionally poor and negatively affect plant productivity. In our experiment, we found *P. australis* grown in dredge soils had an average 44% lower biomass relative to those in healthy soils.

### Suitability of dieback soils

Our study revealed that dieback and healthy soils were chemically different. It is uncertain whether the chemical differences reported between dieback and healthy soils are the cause for, or the consequence of, *P. australis* dieback. However, our healthy sites occurred in the same watershed as dieback sites, often along the same channel and at similar water depths. The high input of water in the MRD makes it unlikely that pollutants would differentially accumulate to the degree reported herein. As such, it is most plausible that the differences in soil chemistry between healthy and dieback sites are a consequence of dieback and indirectly by whatever caused that dieback.

We observed higher concentrations of different groups of compounds in dieback areas compared to healthy areas, including macronutrients (K, Mg and S)^46^, trace metals (Fe, Mn, and Zn), and organic matter content (% OM, % C and % N). Among the myriad of factors associated with dieback, the accumulation of toxins may impair a broad variety of the plant’s metabolic and cellular processes, including internal aeration, photosynthesis, ion absorption, and cell membrane integrity, via blockages by callus formation^18^. Although *P. australis* is commonly used for phytoremediation due to its high tolerance to metals^47^, Furtig et al.^26^ suggested that toxic concentrations of Cu and Fe (Cu^2+^ ≤ 40 µM, Fe^2+^ ≥ 1 mM) can potentially contribute to *P. australis* dieback by impairing root functions (e.g., nutrient uptake, root development and growth). Our results showed dieback soils were above the Fe threshold during both seasons but were highest in the Winter.

Similarly, sulfide toxicity may lead to reductions in root growth and death of roots and rhizomes^12^. Additionally, phytotoxins may cause *P. australis* to become more susceptible to biotic stressors, such as herbivory or the infection of viruses and pathogenic bacteria and fungi^15,23,25,48^ and more prone to mechanical damage^24^. Although we did not measure soil sulfide concentrations in our study, Winter and Spring dieback soils had 75% and 56% greater concentrations of total sulfur than healthy soils; most of which is present as organic sulfur^49^. Further studies are needed that focus on phytotoxins in the MRD, particularly how they might interact with other factors such as water depth, redox potential, and biotic stressors to affect plant fitness and functional traits.

In both seasons, the higher % OM (also % C and % N) in dieback soils than healthy soils may be attributed to the loss of vegetation leading to increased root mortality, decomposition of root tissues and rapid peat collapse^50^. According to Li et al.^51^ and Li et al.^19^, who studied *P. australis* dieback in the Yangtze River Estuary in China, the C and N contents in tissues of dieback *P. australis* were significantly greater than those in healthy *P. australis*. In that study, *P. australis* stems of dieback sites decomposed faster than stems of healthy sites. Anoxic conditions that arise from flood exposure and eutrophication can also be induced by organic matter accumulation^52,53^; however, we could not address this aspect of eutrophication because we conducted our experiments in pots grown in shallow trays or pools where oxygen levels were likely consistently high. In the future, it would be beneficial to monitor how long it takes the soil chemistry of dieback sites to return to levels comparable to sites that have exhibited no dieback.

### *Phragmites australis* lineage and response to soil condition

In support of prediction 2, we did find that some aspect of the chemistry of dieback soils is harmful to *P. australis* growth. Evidence from other field studies suggests that the Delta lineage is more susceptible to dieback and less tolerant of environmental stresses (e.g., flooding, fertilizer, salinity, scale insects)^10,21,22^. In contrast, the EU lineage's invasion across North America has been linked to its superior competitive ability, nutrient assimilation efficiency, adaptability, and herbivore resistance^54–56^. However, we did not find support for prediction 4 that the Delta lineage would be more susceptible to dieback soils or prediction 5 that the EU lineage would outperform the other lineages on dieback soils. In both experiments, Delta and EU exhibited similar biomass reductions when grown in dieback as compared to healthy soils; however, the proportional reduction in biomass was greater in the Spring–Summer experiment; but not significant. Although belowground biomass responses to dieback soils are unknown, several studies suggest that soil toxins can affect *P. australis* root growth, morphology and physiology^55^. Thus, our focus solely on aboveground plant parts may underestimate the impacts of dieback soils on the whole plant.

As predicted (prediction 3), we confirmed that nutrient-poor dredge soils yielded the lowest plant biomass for both Delta (5.93 ± 1.72) and EU (5.04 ± 1.48; Fig. 2), although the difference was only significant for the Delta lineage. The considerably lower biomass implies that characteristics of fine-grained dredged sediments, such as accelerated decomposition rates, lower water holding capacity, and poor nutrient retention may be problematic for plant growth and productivity^37^. However, under resource limited conditions, including low-nutrient soils, plants can compensate for biomass loss by allocating biomass production to belowground parts^57^. We suggest that a longer duration study and/or the inclusion of other plant fitness metrics (e.g., seed production, rhizome growth) will help illuminate the overall health of *P. australis*.

### Constructed wetlands and restoration of the MRD

To offset further wetland loss of the MRD, the Coastal Protection and Restoration Authority (CPRA) of Louisiana has engaged in the creation and restoration of degraded marsh habitats via deposition of dredge sediments^38^. The introduction of dredge sediments, composed primarily of silts and clays, increases marsh surface elevation and reduces flood stress for colonizing plants^58,59^. Our study shows that at least in two dredge sites, many nutrients and metals, including Ca, Cu, Fe, K, Mg, Mn, Na, P, Zn, % OM, % C, and % N occur at low levels relative to healthy *P. australis* marsh sites. Despite reduced growth on these soils, our field observations at dredge sites confirm the natural colonization of *P. australis.* In a brackish marsh in the Barataria Basin of Southeast Louisiana, Howard et al.^60^ demonstrated that transplanted *P. australis* to dredge sites could rapidly spread within two-years. However, further studies are needed to estimate the extent of expansion and the time it would take for *P. australis* to achieve substantial vegetative cover in these newly constructed sites along the MRD.

Dieback syndrome is a recent and widespread problem in the MRD^9,10,21^. Recovery of *P. australis* in the MRD may occur slowly on its own but active restoration plans with more stress-tolerant and fast-growing lineages of *P. australis*, fertilizer addition to nutrient deprived dredged sediments, and/or control of herbivory by the Roseau cane scale or nutria (*Myocastor coypus* (Kerr)) may be needed to expedite reestablishment of ecological and societal services provided by this foundational plant species. Because these services are likely proportional to the biomass and productivity of *P. australis,* extensive dieback of reed stands will severely impair the efficacy of these ecosystem services^39^. The upside of our study’s findings is that soils in areas of dieback are only modestly unsuitable (16–23% reduced biomass relative to healthy soils) and all three lineages of *P. australis* are capable of regrowing on these soils. What we do not know are the long-term effects of these soils on plant growth, reproduction and ability to compete against other aquatic plant species that are less capable of stabilizing marsh soils^61,62^. Unfortunately, our observations are that for many areas where *P. australis* has died off, they are now occupied by invasive floating aquatics. Whether this change in plant community composition is permanent remains to be seen. However, it is possible that the factors that induced dieback in the field may be temporarily active and that the removal of such factors may restore suitable conditions for recovery^63,64^, but if elevation is lost to erosion and subsidence prior to vegetation establishment, it may impede recovery in some areas^21^ We suggest that long-term monitoring of dieback, dredge, and healthy sites will provide comprehensive data to understand patterns of dieback in the lower Mississippi River Delta.

### Supplementary Information

Supplementary Information.

## Data Availability

The data for this study are available from the corresponding author on reasonable request.
