# Supplementary material for: Dieback and dredge soils of Phragmites australis in the Mississippi River Delta negatively impact plant biomass
Source: Sci Rep. 2024 Jan 22;14:1935. doi: 10.1038/s41598-024-52488-4 (PMC10803353; doi:10.1038/s41598-024-52488-4)
Supplement: Supplementary file 1 — Supplementary Information. [file 41598_2024_52488_MOESM1_ESM.docx]

**Supplementary Information**

**Dieback and dredge soils of *Phragmites australis* in the Mississippi River Delta negatively impact plant biomass**

Herie Lee^a^, Rodrigo Diaz^b^ and James T. Cronin^a*^

**Corresponding author*:

James T. Cronin: Department of Biological Sciences, Louisiana State University, 202 Life Sciences Building, Baton Rouge, LA 70803, USA. Tel: 225-578-7218; E-mail address: jcronin@lsu.edu. ORCID #: 0000-0001-7111-6412

^a^ Department of Biological Sciences, Louisiana State University, Baton Rouge, LA, USA 70803

^b^ Department of Entomology, Louisiana State University, Baton Rouge, LA, USA 70803

**Table S1**. Origin and soil type designations for soil surveys (Winter and Spring) and Winter-Spring and Spring-Summer Experiment.

| Season | Soil Type | Latitude | Longitude |
| --- | --- | --- | --- |
| **a) Winter Soil Survey** | Healthy | 29.09262 | -89.21034 |
|  | Healthy | 29.08813 | -89.22776 |
|  | Healthy | 29.12298 | -89.23856 |
|  | Dieback | 29.08304 | -89.21557 |
|  | Dieback | 29.12079 | -89.25319 |
|  | Dieback | 29.11735 | -89.20336 |
|  |  |  |  |
| **b) Spring Soil Survey** | Healthy | 29.103837 | -89.220137 |
|  | Healthy | 29.083732 | -89.247022 |
|  | Dieback | 29.082775 | -89.214178 |
|  | Dieback | 29.117257 | -89.203218 |
|  | Dredge | 29.137427 | -89.217168 |
|  | Dredge | 29.137412 | -89.217142 |

**Table S2**. Overview of soil testing methods conducted by the LSU AgCenter Soil testing and Plant Analysis Lab.

| Soil Test | Extractant | Conditions | Analysis | Reference |
| --- | --- | --- | --- | --- |
| Phosphorous, Potassium, Calcium,  Magnesium, Sodium,  Sulfur,  Copper,  Zinc | Mehlich 3 | 2 g soil/20 mL solution, 5 min shaking  (3.75 M NH4F – 0.25 M EDTA NH4NO3, CH3COOH, and HNO3) | ICP | Mehlich, 1984 |
| pH | Water | 10 g soil / 10 mL deionized H2O 2 hr. equilibration | pH meter + electrode | McLean, 1982 |
| Organic Matter | 1 g soil / 10 mL 1 N K2Cr2O7 + 20 mL conc. H2SO4 (wait 2 hours) + 90 mL H2O, 16 hr. equilibration | 1 g soil / 10 mL 1 N K2Cr2O7 + 20 mL conc. H2SO4 (wait 2 hours) + 90 mL H2O, 16 hr. equilibration | Nelson and Sommer, 1982 | Nelson and Sommer, 1982 |
| Manganese, Iron, Copper, Zinc | DTPA | 10 g soil / 20 mL pH 7.3, 0.005 M DTPA, 2 hr. shaking | ICP | Baker and Amacher, 1982 |
| Aluminum | BaCl2 / NH4Cl | 2 g soil / 20 mL 0.1 M BaCl2 / NH4Cl, 15 min shaking | ICP | Barnhisel and Bertsch, 1982 |
| % C and % N |  | 0.25 g soil | LECO Carbon/Nitrogen Dumas Analyzer. | Dumas Dry-Combustion |

**Table S3**. Lineage, location of origin and coordinates for *P. australis* populations used in (**a**) Winter-Spring and (**b**) Spring-Summer experiments.

| Code | Lineage | Location of Origin | Latitude | Longitude |
| --- | --- | --- | --- | --- |
| **(a) Winter-Spring Experiment** | | | | |
| PLM | Delta | Lower Mississippi River Delta | 29.13 | -89.23 |
| Earl3 | Delta | Lower Mississippi River Delta | 29.17649 | -89.28638 |
| Ear4 | Delta | Lower Mississippi River Delta | 29.05301 | -89.3327 |
| EU3 | EU | Plaquemines Parish, LA, USA | 29.14991 | -89.19962 |
| HI | Gulf | High Island, TX | 29.55194 | -94.389531 |
| SAU | Gulf | Bayou Sauvage NWR | 30.06676 | -89.8273 |
| **(b) Spring-Summer Experiment** | | | | |
| Earl3 | Delta | Lower Mississippi River Delta | 29.17649 | -89.28638 |
| Ear4 | Delta | Lower Mississippi River Delta | 29.05301 | -89.3327 |
| TELM | EU | Missouri | 38.42155 | -90.34016 |
| EU3 | EU | Plaquemines Parish, LA, USA | 29.14991 | -89.19962 |


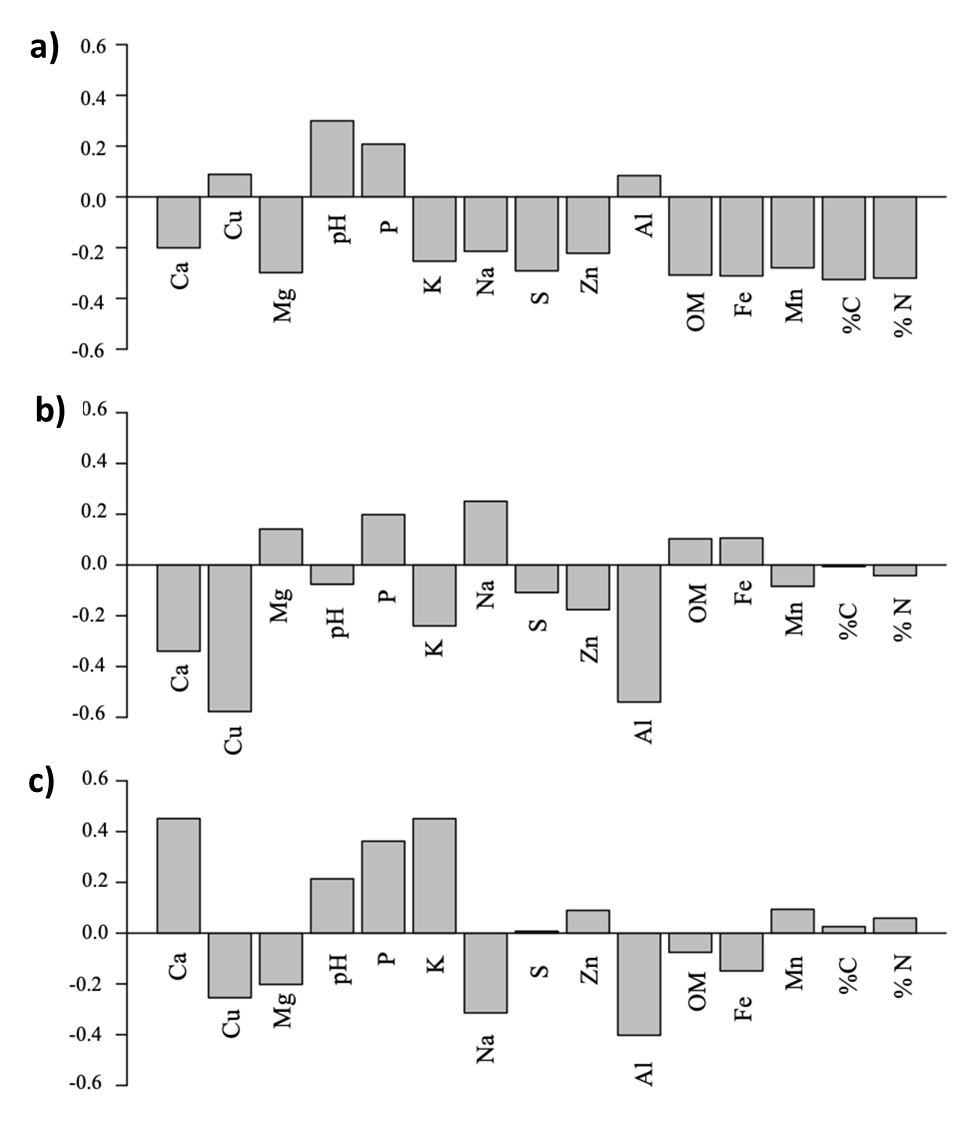
 **Figure S1**. The individual loadings of soil chemical properties for (**a**) PC1, (**b**) PC2 and (**c**) PC3 from the principal component analysis of Winter Survey.

**
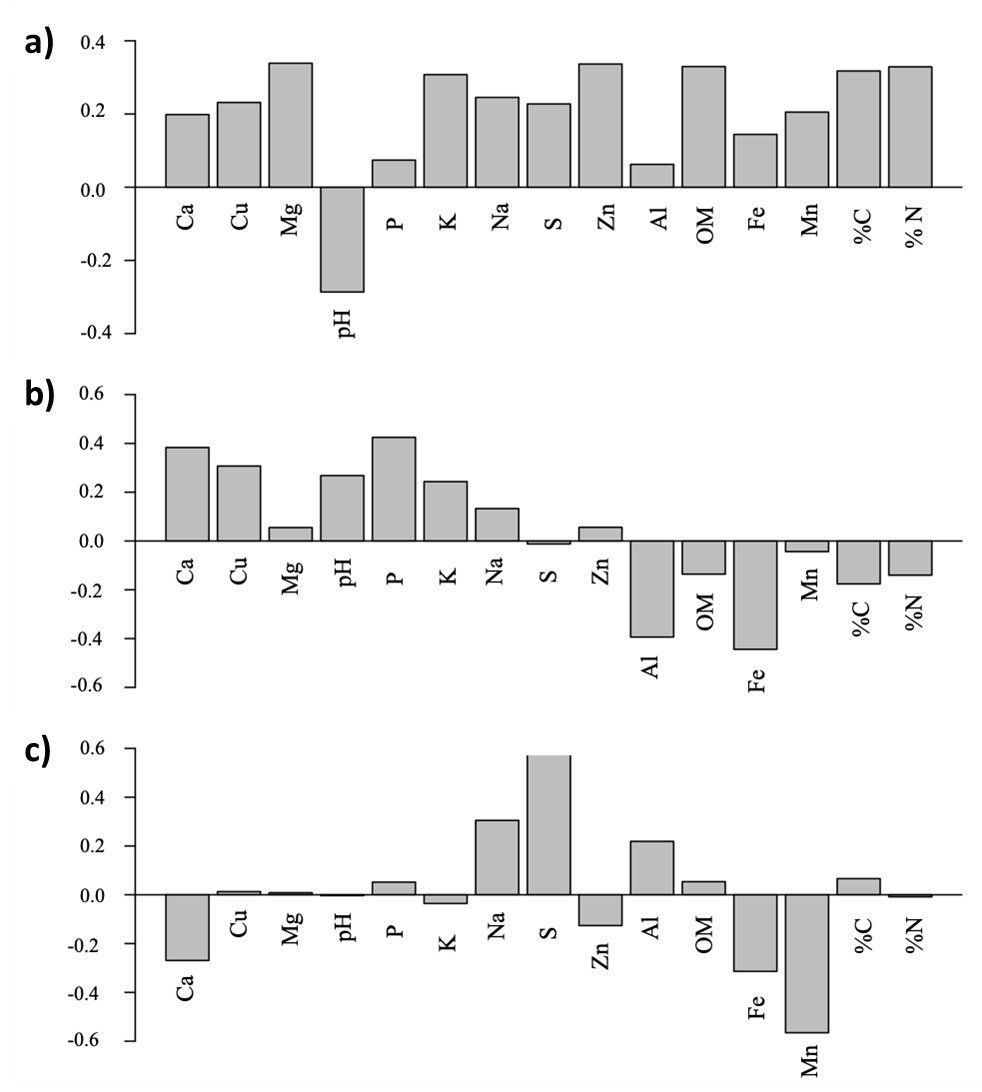
Fig. S2**. The individual loadings of soil chemical properties for (**a**) PC1, (**b**) PC2 and (**c**) PC3 from the principal component analysis of Spring Survey.

**Table S4**. Correlation matrix (Pearson’s product moment correlation, *R*) of correlations between soil chemical properties for the soils in the (**a**) Winter and (**b**) Spring surveys. Values in bold indicate strong correlations (critical value > 0.80) *** *P* ≤ 0.001, ** 0.001 < *P* < 0.01, * 0.01 < *P* ≤ 0.05.

| **a)** Winter Survey | | | | | | | | | | | |  |  |  |  |  |
| --- | --- | --- | --- | --- | --- | --- | --- | --- | --- | --- | --- | --- | --- | --- | --- | --- |
|  | Ca | Cu | Mg | pH | P | K | Na | S | Zn | Al | %OM | | Fe | Mn | %C | %N |
| Ca | 1.00 |  |  |  |  |  |  |  |  |  |  | |  |  |  |  |
| Cu | -0.24 | 1.00 |  |  |  |  |  |  |  |  |  | |  |  |  |  |
| Mg | 0.70** | -0.71** | 1.00 |  |  |  |  |  |  |  |  | |  |  |  |  |
| pH | -0.72** | 0.65** | -**0.99***** | 1.00 |  |  |  |  |  |  |  | |  |  |  |  |
| P | -**0.82***** | 0.31 | -**0.86***** | **0.87***** | 1.00 |  |  |  |  |  |  | |  |  |  |  |
| K | **0.96***** | -0.45 | **0.85***** | -**0.86***** | -**0.86***** | 1.00 |  |  |  |  |  | |  |  |  |  |
| Na | 0.53* | -0.79*** | **0.95***** | -**0.91***** | -0.75** | 0.69** | 1.00 |  |  |  |  | |  |  |  |  |
| S | **0.85***** | -0.55* | **0.95***** | -**0.96***** | -**0.93***** | **0.94***** | **0.84***** | 1.00 |  |  |  | |  |  |  |  |
| Zn | 0.79*** | -0.42 | **0.87***** | -**0.91***** | -**0.80***** | **0.90***** | 0.69** | **0.89***** | 1.00 |  |  | |  |  |  |  |
| Al | -0.27 | **0.99***** | -0.68** | 0.62* | 0.27 | -0.47 | -0.74** | -0.53* | -0.42 | 1.00 |  | |  |  |  |  |
| %OM | 0.74** | -0.71** | **1.00***** | -**0.99***** | -**0.86***** | **0.88***** | **0.94***** | **0.95***** | **0.88***** | -0.68** | 1.00 | |  |  |  |  |
| Fe | 0.72** | -0.70** | **0.99***** | -**0.99***** | -**0.86***** | **0.87***** | **0.93***** | **0.96***** | **0.88***** | -0.67** | **0.99***** | | 1.00 |  |  |  |
| Mn | **0.85***** | -0.58* | **0.94***** | -**0.94***** | -**0.92***** | **0.94***** | **0.85***** | **0.99***** | **0.84***** | -0.57 | **0.95***** | | **0.96***** | 1.00 |  |  |
| %C | **0.80***** | -0.64** | **0.98***** | -**0.99***** | -**0.89***** | **0.92***** | **0.89***** | **0.98***** | **0.91***** | -0.62* | **0.99***** | | **0.99***** | **0.98***** | 1.00 |  |
| %N | **0.82***** | -0.61* | **0.97***** | -**0.98***** | -**0.90***** | **0.94***** | **0.86***** | **0.99***** | **0.92***** | -0.60* | **0.98***** | | **0.98***** | **0.98***** | **1.00***** | 1.00 |

| **b)** Spring Survey | | | | | | | | | | | |  |  |  |  |  |
| --- | --- | --- | --- | --- | --- | --- | --- | --- | --- | --- | --- | --- | --- | --- | --- | --- |
|  | Ca | Cu | Mg | pH | P | K | Na | S | Zn | Al | %OM | | Fe | Mn | %C | %N |
| Ca | 1.00 |  |  |  |  |  |  |  |  |  |  | |  |  |  |  |
| Cu | 0.29 | 1.00 |  |  |  |  |  |  |  |  |  | |  |  |  |  |
| Mg | 0.65** | 0.08 | 1.00 |  |  |  |  |  |  |  |  | |  |  |  |  |
| pH | -0.12 | -0.11 | -0.72 | 1.00 |  |  |  |  |  |  |  | |  |  |  |  |
| P | 0.20 | 0.71** | -0.12 | 0.17 | 1.00 |  |  |  |  |  |  | |  |  |  |  |
| K | **0.81***** | 0.37 | **0.88***** | -0.53* | 0.24 | 1.00 |  |  |  |  |  | |  |  |  |  |
| Na | 0.45 | -0.07 | 0.79 | -0.40 | 0.04 | 0.74 | 1.00 |  |  |  |  | |  |  |  |  |
| S | 0.17 | 0.14 | 0.65** | -0.55* | -0.14 | 0.55* | 0.67** | 1.00 |  |  |  | |  |  |  |  |
| Zn | 0.45 | **0.80***** | 0.52* | -0.58* | 0.56* | 0.68** | 0.27 | 0.28 | 1.00 |  |  | |  |  |  |  |
| Al | -0.22 | -0.20 | 0.17 | -0.29 | -0.28 | -0.12 | 0.13 | 0.26 | 0.00 | 1.00 |  | |  |  |  |  |
| %OM | 0.33 | 0.36 | 0.79 | -**0.90***** | 0.09 | 0.70** | 0.49* | 0.62** | 0.78 | 0.28 | 1.00 | |  |  |  |  |
| Fe | -0.06 | -0.20 | 0.35 | -0.60* | -0.45 | 0.13 | 0.14 | 0.03 | 0.37 | 0.56* | 0.48* | | 1.00 |  |  |  |
| Mn | 0.38 | 0.37 | 0.55* | -0.48* | 0.08 | 0.49* | 0.25 | 0.04 | 0.59* | 0.00 | 0.50* | | 0.50* | 1.00 |  |  |
| %C | 0.24 | 0.27 | 0.76 | -**0.90***** | 0.10 | 0.64** | 0.54* | 0.58* | 0.72 | 0.31 | **0.97***** | | 0.53* | 0.50* | 1.00 |  |
| %N | 0.33 | 0.35 | 0.78 | -**0.92***** | 0.11 | 0.70** | 0.48* | 0.56* | 0.78 | 0.26 | **0.99***** | | 0.52* | 0.55* | **0.98***** | 1.00 |

**Table S5**. Separate *t*-test results for the difference in soil chemicals between healthy and dieback sites from the Winter Survey. Significance, denoted by * adjacent to the *P*-value, was determined using Bonferroni-corrected values of α.

| Variable | Soil Type | *n* | Mean | SE | *t* | *P*-value |
| --- | --- | --- | --- | --- | --- | --- |
| Al (ppm) | Dieback | 8 | 0.58 | 0.10 | -1.22 | 0.308 |
|  | Healthy | 7 | 2.58 | 1.64 |  |  |
| Ca (ppm) | Dieback | 8 | 3992.48 | 297.21 | 0.04 | 0.968 |
|  | Healthy | 7 | 4017.69 | 284.89 |  |  |
| Cu (ppm) | Dieback | 8 | 1.16 | 0.11 | -1.40 | 0.261 |
|  | Healthy | 7 | 1.89 | 0.53 |  |  |
| Fe (ppm) | Dieback | 8 | 160.82 | 16.12 | 5.33 | **<0.001*** |
|  | Healthy | 7 | 71.06 | 4.93 |  |  |
| K (ppm) | Dieback | 8 | 261.66 | 28.61 | 0.90 | 0.414 |
|  | Healthy | 7 | 231.66 | 23.37 |  |  |
| Mg (ppm) | Dieback | 8 | 1174.23 | 53.25 | 7.47 | **<0.001*** |
|  | Healthy | 7 | 620.78 | 52.18 |  |  |
| Mn (ppm) | Dieback | 8 | 127.84 | 52.49 | 1.85 | 0.145 |
|  | Healthy | 7 | 30.27 | 6.28 |  |  |
| Na (ppm) | Dieback | 8 | 426.77 | 51.71 | 2.98 | **0.027*** |
|  | Healthy | 7 | 230.48 | 28.65 |  |  |
| % OM | Dieback | 8 | 5.77 | 0.34 | 5.21 | **<0.001*** |
|  | Healthy | 7 | 3.07 | 0.36 |  |  |
| P (ppm) | Dieback | 8 | 27.76 | 1.42 | -2.21 | 0.085 |
|  | Healthy | 7 | 34.20 | 2.46 |  |  |
| pH | Dieback | 8 | 5.80 | 0.27 | -6.55 | **<0.001*** |
|  | Healthy | 7 | 7.40 | 0.06 |  |  |
| S (ppm) | Dieback | 8 | 1121.41 | 404.80 | 2.19 | 0.106 |
|  | Healthy | 7 | 280.07 | 57.66 |  |  |
| Zn (ppm) | Dieback | 8 | 9.07 | 0.57 | 1.94 | 0.112 |
|  | Healthy | 7 | 7.28 | 0.58 |  |  |
| % C | Dieback | 8 | 5.51 | 0.80 | 3.84 | **0.011*** |
|  | Healthy | 7 | 2.19 | 0.30 |  |  |
| % N | Dieback | 8 | 0.51 | 0.08 | 3.69 | **0.014*** |
|  | Healthy | 7 | 0.19 | 0.02 |  |  |

**Table S6**. Analysis of variance results for the effect of different soil types (healthy, dieback, dredge) on separate soil chemical characteristics from the Spring survey. Post-Hoc Tukey HSD tests were used to assess differences between treatment pairs. For each variable, means with different letters are significantly different (*P* ≤ 0.05). Significance denoted by * adjacent to the *P*-value.

| Variable | Soil Type | Mean | SE | *F* | *P*-value |
| --- | --- | --- | --- | --- | --- |
| Al (ppm) | Healthy | 0.64^a^ | 0.05 | 1.51 | 0.25 |
|  | Dieback | 0.93^a^ | 0.07 |  |  |
|  | Dredge | 0.79^a^ | 0.18 |  |  |
| Ca (ppm) | Healthy | 3546.70^a^ | 366.1 | 12.30 | **0.001*** |
|  | Dieback | 2412.31^b^ | 199.37 |  |  |
|  | Dredge | 1653.18^b^ | 218.48 |  |  |
| Cu (ppm) | Healthy | 1.57^a^ | 0.26 | 5.983 | **0.012*** |
|  | Dieback | 1.09^ab^ | 0.14 |  |  |
|  | Dredge | 0.74^b^ | 0.03 |  |  |
| Fe (ppm) | Healthy | 64.3^ab^ | 7.52 | 4.41 | **0.033*** |
|  | Dieback | 114.01^a^ | 20.04 |  |  |
|  | Dredge | 51.11^b^ | 15.83 |  |  |
| K (ppm) | Healthy | 219.87^a^ | 21.45 | 14.51 | **<0.001*** |
|  | Dieback | 209.63^b^ | 23.83 |  |  |
|  | Dredge | 82.47^b^ | 13.52 |  |  |
| Mg (ppm) | Healthy | 617.43^a^ | 94.82 | 31.87 | **<0.001*** |
|  | Dieback | 823.07^a^ | 76.96 |  |  |
|  | Dredge | 279.04^b^ | 23.55 |  |  |
| Mn (ppm) | Healthy | 136.84^ab^ | 36.66 | 5.33 | **0.02*** |
|  | Dieback | 153.66^a^ | 28.40 |  |  |
|  | Dredge | 42.25^b^ | 12.68 |  |  |

*(table cont’d.)*

| Variable | Soil Type | Mean | SE | *F* | *P*-value |
| --- | --- | --- | --- | --- | --- |
| Na (ppm) | Healthy | 282.87^a^ | 66.58 | 2.072 | 0.161 |
|  | Dieback | 381.88^a^ | 87.75 |  |  |
|  | Dredge | 192.60^a^ | 29.1 |  |  |
| % OM | Healthy | 2.89^a^ | 0.23 | 35.75 | **<0.001*** |
|  | Dieback | 5.29^b^ | 0.61 |  |  |
|  | Dredge | 0.73^c^ | 0.12 |  |  |
| P (ppm) | Healthy | 72.89^a^ | 8.75 | 1.973 | 0.174 |
|  | Dieback | 54.10^a^ | 6.39 |  |  |
|  | Dredge | 58.7^a^ | 5.33 |  |  |
| pH | Healthy | 7.43^a^ | 0.06 | 104.20 | **<0.001*** |
|  | Dieback | 6.41^b^ | 0.09 |  |  |
|  | Dredge | 7.71^a^ | 0.04 |  |  |
| S (ppm) | Healthy | 44.70^ab^ | 10.37 | 4.171 | **0.036*** |
|  | Dieback | 100.89^a^ | 29.98 |  |  |
|  | Dredge | 27.68^b^ | 7.02 |  |  |
| Zn (ppm) | Healthy | 7.58^a^ | 0.89 | 15.70 | **<0.001*** |
|  | Dieback | 7.34^a^ | 0.22 |  |  |
|  | Dredge | 3.01^b^ | 0.89 |  |  |
| % C | Healthy | 1.42^a^ | 0.12 | 25.79 | **<0.001*** |
|  | Dieback | 3.04^b^ | 0.42 |  |  |
|  | Dredge | 0.49^c^ | 0.27 |  |  |
| % N | Healthy | 0.05^a^ | 0.01 | 31.77 | **<0.001*** |
|  | Dieback | 0.30^b^ | 0.04 |  |  |
|  | Dredge | 0.17^c^ | 0.00 |  |  |

**Table S7**. Analysis of variance results for the effects of soil type (dieback, healthy), *P. australis* lineage (Delta, EU, Gulf), and the lineage × soil type interaction on **a**) *ln* total stem count per pot and **b**) max stem height for Winter-Spring Experiment and for the effects of soil type (dieback, healthy, dredge), *P. australis* lineage (Delta, EU), and the lineage × soil type interaction on **c**) total stem count per pot and **d**) max stem height for Spring-Summer Experiment. Sources of variation with an * indicate significance (*P* ≤ 0.05).

|  | Sums of Squares | | *df* (num) | *df* (denom) | *F* | *P*-value |
| --- | --- | --- | --- | --- | --- | --- |
| **WINTER-SPRING EXPERIMENT**  **a) *ln* total stem count per pot** | |  | |  |  |  |
| Lineage | 0.38 | 2 | | 2.99 | 1.87 | 0.297 |
| Soil Type | 0.78 | 1 | | 9.5 | 7.7 | **0.021*** |
| Lineage × Soil Type | 0.12 | 2 | | 225.63 | 0.58 | 0.562 |
|  |  |  | |  |  |  |
| **b) Max stem height** | | | | | | |
| Lineage | 0.15 | 2 | | 3 | 2.16 | 0.263 |
| Soil Type | 0.01 | 1 | | 228.26 | 0.19 | 0.66 |
| Lineage × Soil Type | 0.13 | 2 | | 228.3 | 1.91 | 0.151 |
|  |  |  | |  |  |  |
| **SPRING-SUMMER EXPERIMENT**  **c) Total stem count per pot** | |  | |  |  |  |
| Lineage | 2.67 | 1 | | 1.77 | 0.16 | 0.733 |
| Soil Type | 405.9 | 2 | | 22.86 | 12.1 | **< 0.001*** |
| Lineage × Soil Type | 15.06 | 2 | | 122.64 | 0.45 | 0.639 |
|  |  |  | |  |  |  |
| **d) Max stem height** | | | | | | |
| Lineage | 818.5 | 1 | | 1.68 | 3.66 | 0.22 |
| Soil Type | 784.74 | 2 | | 17.51 | 1.75 | 0.202 |
| Lineage × Soil Type | 1579.58 | 2 | | 113.07 | 3.53 | **0.033*** |


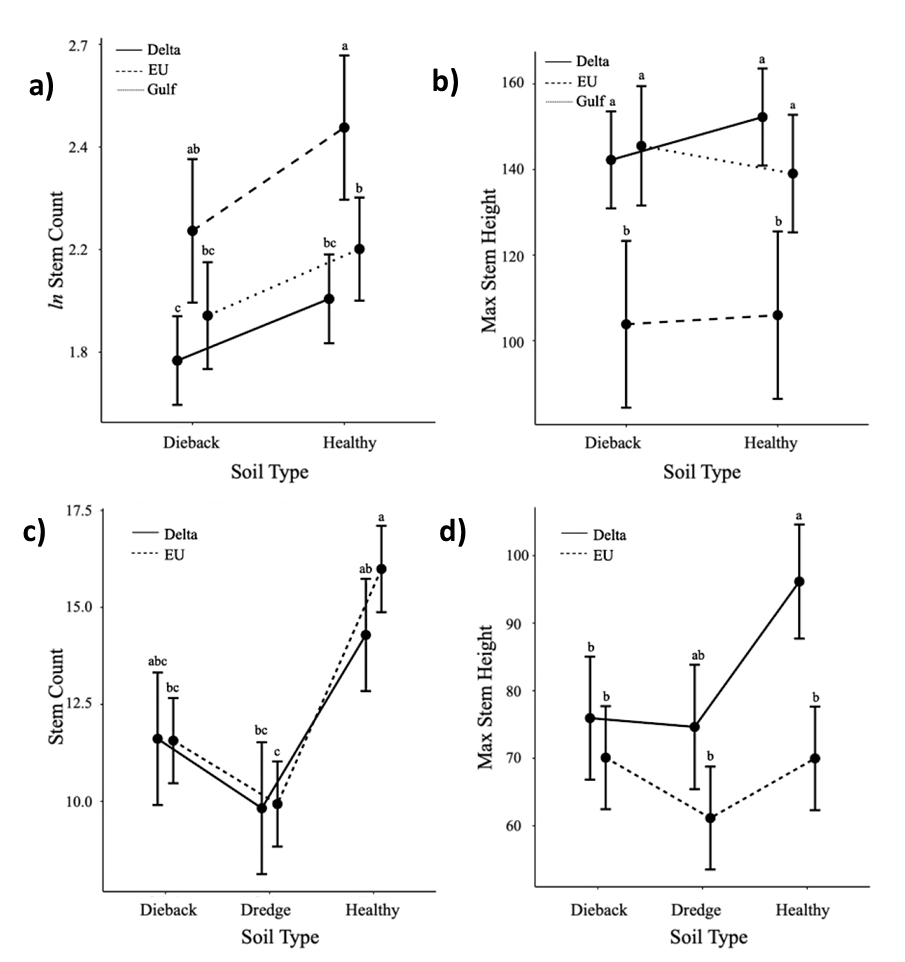


**Fig. S3**. The effects of soil type (dieback, healthy) and *P. australis* lineage (Delta, EU, Gulf) on **a**) *ln* total stem count per pot and **b**) max stem height (cm) for Winter-Spring Experiment and the effects of soil type (dieback, healthy, dredge) and *P. australis* lineage (Delta, EU) on **c**) total stem count per pot and **d**) max stem height (cm) for Spring-Summer Experiment. Reported values are marginal means ± SE. Different letters between groups indicate significant differences at *P* ≤ 0.05.
